# TRIM38 Suppresses the Progression of Colorectal Cancer via Enhancing CCT6A Ubiquitination to Inhibit the MYC Pathway

**DOI:** 10.1002/advs.202411285

**Published:** 2025-03-06

**Authors:** Yue Zhang, Xinyu Tan, Lu Wang, Dongjian Ji, Chuan Zhang, Wen Peng, Renzhong Zhu, Xiaowei Wang, Jiahui Zhou, Yifei Feng, Yueming Sun

**Affiliations:** ^1^ Department of General Surgery The First Affiliated Hospital of Nanjing Medical University Nanjing Jiangsu 210029 P. R. China; ^2^ The First School of Clinical Medicine Nanjing Medical University Nanjing 210029 P. R. China; ^3^ Colorectal Institute of Nanjing Medical University Nanjing 210029 P. R. China; ^4^ Jiangsu Province Engineering Research Center of Colorectal Cancer Precision Medicine and Translational Medicine Nanjing 210029 P. R. China; ^5^ Institute of Translational Medicine Medical College Yangzhou University Yangzhou Jiangsu 225000 P. R. China; ^6^ The Affiliated Suzhou Hospital of Nanjing Medical University Suzhou Municipal Hospital, Gusu School Nanjing Medical University Suzhou Jiangsu 215000 P. R. China

**Keywords:** CCT6A, colorectal cancer, MYC, TRIM38, ubiquitination

## Abstract

Emerging evidence reveals the pivotal function of tripartite motif protein (TRIM) in colorectal cancer (CRC). However, the precise function of TRIM38 and its underlying mechanism in CRC remains to be elucidated, especially regarding its putative ubiquitination function. Here, it is identified that TRIM38 is downregulated in CRC tissues by DNA hypermethylation of its promoter. Further analysis demonstrates that decreased TRIM38 is correlated with unfavorable clinical features and poor prognosis. Moreover, TRIM38 functions as a tumor suppressor by inhibiting cell proliferation, metastasis, and AOM/DSS‐induced tumorigenesis in CRC cells. Mechanistically, TRIM38 binds to the substrate protein CCT6A, leading to the degradation and K48‐linked ubiquitination of CCT6A at the K127/K138 residues. The elevation of CCT6A protein level caused by TRIM38 downregulation diminishes the degradation of c‐Myc protein, thereby activating the MYC pathway. The study elucidates a novel mechanism of TRIM38/CCT6A/c‐Myc axis regulating CRC, potentially offering a new therapeutic target for its treatment.

## Graphical abstract

This study reveals the tumor‐suppressive role of TRIM38 in colorectal cancer (CRC). TRIM38 is downregulated through DNA hypermethylation and correlates with unfavorable clinical features and poor prognosis. Mechanistically, TRIM38 inhibits CRC progression by promoting K48‐linked ubiquitination of CCT6A, leading to the destabilization of c‐Myc. This work highlights the TRIM38/CCT6A/c‐Myc axis as a novel therapeutic target in CRC.

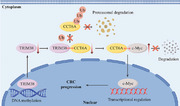

## 1 Introduction

Colorectal cancer (CRC), the third most prevalent cancer and the second leading cause of cancer‐related deaths worldwide, continues to raise a critical public health concern.^[^ ^1^ ^]^ Although comprehensive treatment strategies, including radical resection, radiotherapy, chemotherapy, targeted therapy, and immunotherapy, have made progress in recent years, the prognosis for patients with advanced CRC remains unsatisfactory.^[^ ^2^, ^3^ ^]^ The primary reason for this is that the precise mechanism of CRC progression remains unknown. Consequently, it is of the utmost importance to further explore the potential molecular mechanisms and develop new therapeutic targets for CRC.

The tripartite motif protein (TRIM) is composed of a RING domain, one or two B‐box motifs, and a coiled–coil (CC) domain followed by highly variable carboxyl‐terminal domains from the N‐terminal to the C‐terminal.^[^ ^4^ ^]^ To date, an excess of 80 TRIM proteins are recorded and classified into 11 categories according to the variable carboxyl‐terminal domains.^[^ ^5^ ^]^ TRIM proteins are engaged in a broad range of biological processes and cellular activities, such as immune response, inflammation, glucose metabolism, protein interaction, cell death, and cell cycle.^[^ ^6^, ^7^, ^8^ ^]^ A variety of diseases have been reported to be closely related to the dysregulation of TRIM proteins.^[^ ^8^, ^9^, ^10^ ^]^ In recent years, the role of TRIM in solid tumors has garnered considerable attention due to its critical involvement in tumorigenesis and cancer development through a variety of molecular mechanisms, including transcriptional and post‐translational regulation, epithelial‐mesenchymal transition (EMT) and signaling pathways dysregulation.^[^ ^11^, ^12^, ^13^, ^14^ ^]^ Nevertheless, the specific roles of certain TRIM protein members in CRC remain to be further elucidated.

Ubiquitination represents a crucial post‐translational modification that covalently attaches the 76‐amino acid ubiquitin (Ub) protein to substrate proteins.^[^ ^15^ ^]^ This process is prevalent and vital for cellular functions, with its dysregulation potentially contributing to disease onset and progression, particularly in the cancer.^[^ ^16^, ^17^, ^18^ ^]^ Ubiquitination is catalyzed by a three‐enzyme cascade consisting of the E1 Ub‐activating enzyme, the E2 Ub‐conjugating enzyme, and the E3 Ub ligase.^[^ ^15^ ^]^ Wherein the substrate proteins recognized by E3 ligases frequently determine the oncogenic or anti‐cancer roles of ubiquitination in carcinogenesis.^[^ ^19^, ^20^ ^]^ The TRIM protein, regarded as a RING‐type E3 ligase, exerts a crucial regulatory role in cancers via ubiquitination.^[^ ^5^ ^]^ For example, TRIM1 promotes the proliferation and migration of CRC by enhancing HIF1α ubiquitination.^[^ ^21^ ^]^ TRIM36 interacts with FOXA2 and induces its ubiquitination in CRC.^[^ ^11^ ^]^ However, studies regarding the function of TRIM protein as an E3 ligase in CRC are scarce.

The cytosolic chaperonin CCT (chaperonin containing TCP‐1) is a 1 MDa multi‐subunit protein complex, composed of 8 homologous subunits: CCT1, CCT2, CCT3, CCT4, CCT5, CCT6A/B, CCT7, and CCT8.^[^ ^22^, ^23^ ^]^ Each subunit contains three domains: apical, intermediate, and equatorial.^[^ ^24^ ^]^ CCT is essential in mediating the folding of the eukaryotic cytoskeletal protein dependent on ATP and is also named TRiC (TCP‐1 Ring Complex).^[^ ^25^ ^]^ Emerging studies indicate that each subunit exhibits unique functions in tumorigenesis. For example, CCT3 has been demonstrated to facilitate the proliferation and metastasis of malignant tumors, including breast cancer and cervical cancer.^[^ ^26^, ^27^ ^]^ Moreover, the expression levels of both CCT2 and CCT8 may serve as valuable prognostic indicators in CRC.^[^ ^22^, ^28^ ^]^

In this study, we identified that TRIM38 was significantly downregulated in CRC mediated by DNA methylation. Clinical analysis revealed that decreased TRIM38 was correlated with tumor progression and poor prognosis. Further in vivo and in vitro experiments demonstrated that TRIM38 suppressed the cell proliferation, metastasis, and AOM/DSS‐induced tumorigenesis of CRC cells. Mechanically, the downregulated TRIM38 reduced the degradation and ubiquitination of CCT6A, thereby increasing the stability of c‐Myc mediated by CCT6A.

## 2 Results

### 2.1 TRIM38 is Reduced in CRC and Correlated with Adverse Clinical Features

**Figure 1:**
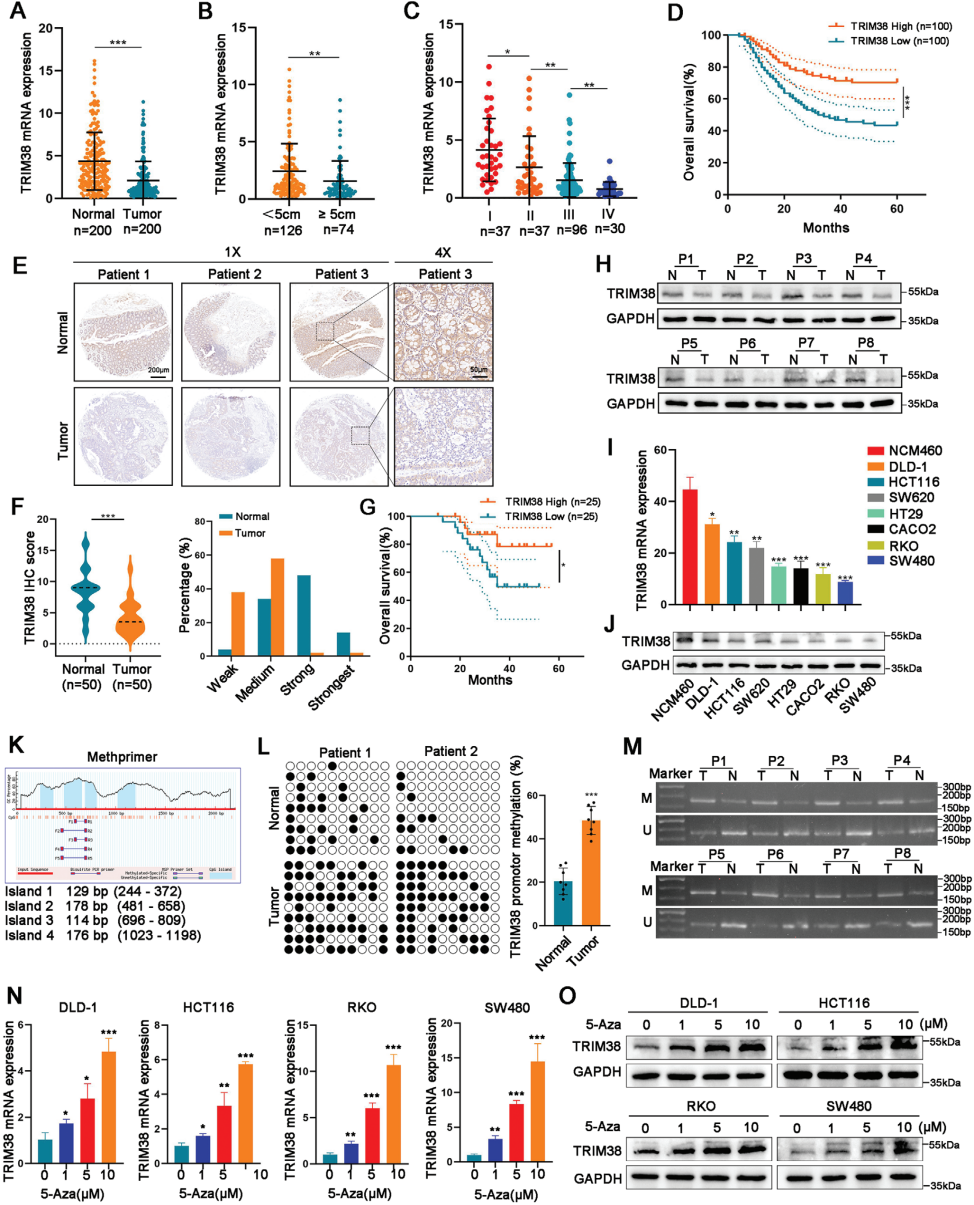
TRIM38 is downregulated in CRC mediated by DNA methylation. A) Relative TRIM38 mRNA expression in 200 CRC tissues and adjacent normal tissues. B,C) Relative TRIM38 mRNA expression in 200 CRC tissues categorized by tumor size and tumor stage. D) Overall survival of 200 CRC patients based on TRIM38 mRNA expression (median method) by Kaplan–Meier analysis. E) IHC‐detected representative images of TRIM38 protein levels in a tissue microarray. F) Analysis of H‐scores and staining degrees of TRIM38 in the tissue microarray. G) Overall survival of 50 CRC patients grouped by the TRIM38 protein level in the tissue microarray by Kaplan–Meier analysis. H) Relative TRIM38 protein levels in 8 pairs of CRC and adjacent normal tissues by western blot. I,J) TRIM38 mRNA expression and protein level in normal colorectal epithelial cells (NCM460) and different CRC cell lines. K) Schematic structure of the TRIM38 CpG island using the Methprimer tool. L) BSP results in tumor and adjacent tissues from 8 CRC patients. M) MSP results in tumor and adjacent tissues from 8 CRC patients. M: methylated, U: unmethylated. N,O) Relative TRIM38 mRNA expression and protein levels in CRC cells following 5‐Aza treatment. Data are presented as mean ± SD from three independent experiments, ^*^ *P* < 0.05, ^**^ *P* < 0.01, ^***^ *P* < 0.001.

We first identified that TRIM38 was significantly downregulated in COAD based on the TCGA database and GEO datasets (GSE106582, GSE20842, GSE41258, GSE21510) (Figure S1A,B, Supporting Information). To further reveal the role of TRIM38 in CRC, we measured the TRIM38 mRNA levels in 200 pairs of tumor tissues and adjacent normal tissues from CRC patients by qRT‐PCR, revealing a significant downregulation of TRIM38 in CRC (**Figure** **1**A), consistent with findings from the TCGA and GEO databases. By analyzing the clinicopathological characteristics, we found that TRIM38 mRNA expression was negatively correlated to tumor size, depth of invasion, liver metastasis as well as tumor stage (**Table** **1** and Figure 1B,C). Moreover, the Kaplan–Meier analysis indicated that reduced TRIM38 mRNA expression in CRC exhibited poor overall survival (Figure 1D), and the multivariate analysis further revealed that TRIM38 was an independent unfavorable prognostic factor for CRC (**Table** **2**). Next, the tissue microarray made from 50 patient samples was used to detect the TRIM38 protein level in CRC. Immunohistochemistry (IHC) exhibited the downregulated TRIM38 protein level in CRC tissues, as reflected by the reduced H‐scores and the weaker degrees of staining obtained (Figure 1E,F). Meanwhile, patients with reduced TRIM38 protein levels have a worse prognosis (Figure 1G). Western blot detecting 8 pairs of CRC and corresponding adjacent normal also indicated a reduction of TRIM38 protein levels in CRC (Figure 1H). Correspondingly, the mRNA and protein level of TRIM38 in CRC cells was significantly reduced compared to those in normal colorectal epithelial cells NCM460 detected by qRT‐PCR and western blot, respectively (Figure 1I,J). Taken together, these results revealed that TRIM38 is reduced in CRC and might play an anti‐cancer role in CRC.

**Table 1:** Analysis of clinical relevance of TRIM38 level in CRC Patients.

Characteristics	TRIM38 level			
	N	high	low	*P* value
Gender				
male	118	62	56	0.388
female	82	38	44	
Age (year)				
≥ 60	125	66	59	0.307
< 60	75	34	41	
Tumor size				
≥ 5cm	74	30	44	0.040^*^
< 5cm	126	70	56	
Depth of invasion				
T1/T2	50	32	18	0.022^*^
T3/T4	150	68	82	
Lymph node metastasis				
Neagtive	87	49	38	0.117
Positive	113	51	62	
Liver metastasis				
Positive	30	8	22	0.006^*^
			
Neagtive	170	92	78
Location				
Rectum	111	53	58	0.477
Colon	89	47	42	

* *P *< 0.05.

**Table 2:** Univariate and multivariate analysis of correlation between prognosis and clinicopathologic parameters in CRC by Cox's regression.

Variable	Univariable analysis		Multivariable analysis	
	HR [95% CI]	P value	HR [95% CI]	*P* value
Sex (male vs female)	0.49 (0.76‐1.79)	0.489	‐	NA
Age (≥60 vs.<60 years)	1.20 (0.77‐1.85)	0.419	‐	NA
Tumor size (≥5cm vs.<5cm)	0.75 (0.49‐1.16)	0.194	‐	NA
Depth of invasion (T1/T2 vs T3/T4)	1.70 (0.98‐2.93)	0.057	‐	NA
Lymph node metastasis (Neagtive vs Positive)	1.70 (1.09‐2.67)	0.021^*^	1.82 (1.13‐2.94)	0.015^*^
Liver metastasis (Positive vs Neagtive)	0.18 (0.11‐0.28)	0.001^*^	0.17 (0.11‐0.29)	0.001^*^
Location (Rectum vs Colon)	0.82 (0.53‐1.26)	0.365	‐	NA
TRIM38 expression (High vs Low)	2.32 (1.48‐3.65)	0.001^*^	1.72 (1.08‐2.75)	0.023^*^

* *P* < 0.05.

### 2.2 TRIM38 is Silenced by Promotor CpG Methylation in CRC

Predictive analysis using MethPrimer identified typical CpG islands in the promoter region of TRIM38 (Figure 1K). Bioinformatics analysis from cBioPortal revealed a negative correlation between the mRNA expression and methylation levels of TRIM38 in CRC (Figure S1C, Supporting Information), suggesting that the silencing of TRIM38 may be mediated by CpG methylation. To validate the hypothesis, we examined the methylation status of TRIM38 in 8 pairs of CRC tissues and matched adjacent normal tissues using Methylation‐specific PCR (MSP) and bisulfite sequencing PCR (BSP), and the results showed that TRIM38 was hypermethylated in CRC tissues (Figure 1L,M). Furthermore, four types of CRC cell lines (DLD‐1, HCT116, RKO, SW480) were treated with different doses of 5‐Aza‐2′‐deoxycytidine (5‐Aza), and the results of qRT‐PCR and western blot showed that the mRNA expression and protein levels of TRIM38 were significantly positively correlated with the dose of 5‐Aza (Figure 1N,O). Therefore, all the findings illustrated that TRIM38 is silenced by promoter CpG methylation in CRC.

### 2.3 TRIM38 Suppresses the Proliferation and Tumorigenesis of CRC Cells In Vitro and In Vivo

**Figure 2:**
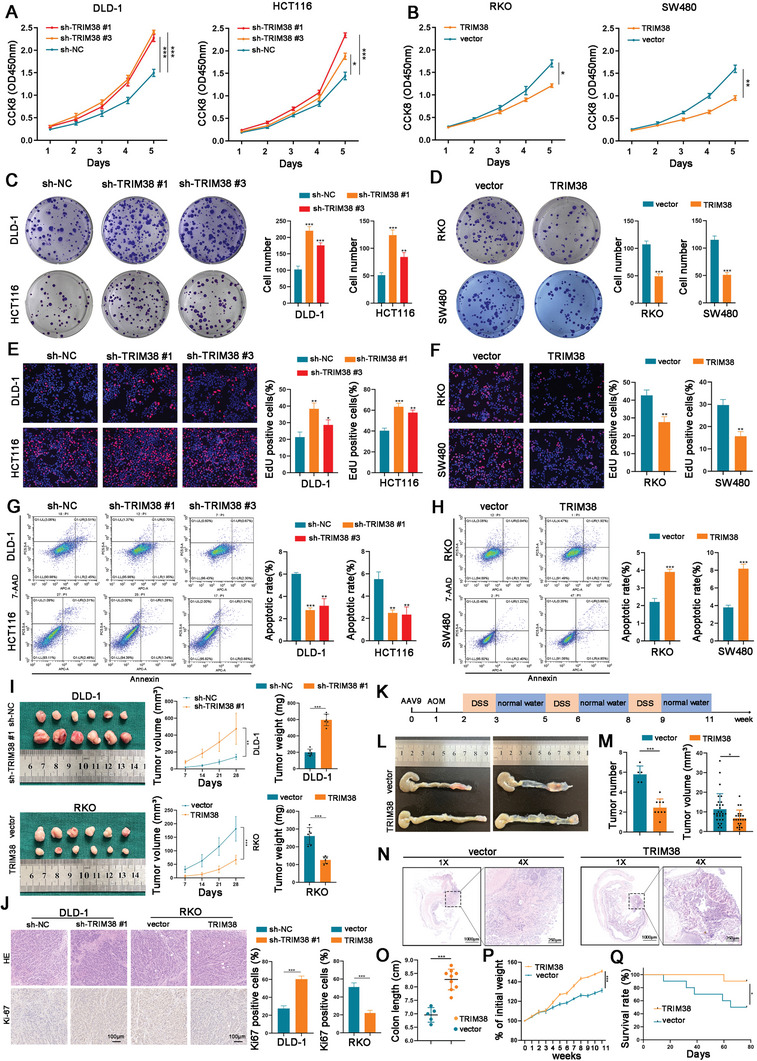
TRIM38 suppresses CRC cell proliferation and tumorigenesis in vitro and in vivo. A,B) CCK‑8 assays were used to detect the impact of TRIM38 silencing or overexpression on CRC cell viability. C,D) Colony formation assays were applied to measure the effect of TRIM38 silencing or overexpression on CRC cell proliferation ability. E,F) EdU assays were performed to assess the effect of TRIM38 silencing or overexpression on CRC cell proliferation. G,H) The role of TRIM38 on CRC cell apoptotic rates was detected by cell apoptosis analysis. I) Photographs of subcutaneous tumors from nude mice in different TRIM38 treatment groups. Tumor volume and tumor weight were measured. J) H&E staining and Ki‐67 expression level measured by IHC in subcutaneous tumors. The percentage of Ki67‐positive cells was quantified. K) Flowchart for the Construction of the AOM/DSS Model in C57/B6 mice. L) Representative images of colons in AAV9‐TRIM38 and AAV9‐vector groups. M) The number of tumors per mouse and tumor volume were quantified. AAV9‐TRIM38: n = 22 tumors from 9 mice. AAV9‐vector: n = 29 tumors from 5 mice. N) H&E staining of colons in AAV9‐TRIM38 and AAV9‐vector groups. O) Colon length statistics in AAV9‐TRIM38 and AAV9‐vector groups. P,Q) The relative body weight changes and survival curves of mice in AAV9‐TRIM38 and AAV9‐vector groups. Data are presented as mean ± SD from three independent experiments, ^*^ *P* < 0.05, ^**^ *P* < 0.01, ^***^ *P* < 0.001.

To investigate the biological function of TRIM38 in CRC, we selected DLD‐1 and HCT116 cells with relatively high TRIM38 expression, as well as RKO and SW480 cells with relatively low TRIM38 expression. Subsequently, we constructed TRIM38 knockdown and TRIM38 overexpression cell lines in the aforementioned cells using lentivirus and plasmid transfection, respectively. The efficiency of TRIM38 knockdown and overexpression was validated (Figures S1D,E and S2C,D, Supporting Information), and sh‐TRIM38 #1 and sh‐TRIM38 #3 were selected for further studies based on their superior efficacy. CCK‐8, colony formation, and EdU assays demonstrated that knocking down TRIM38 significantly promoted cell proliferation of DLD‐1 and HCT116 cells, whereas TRIM38 overexpression exerted an opposing effect on RKO and SW480 cells (**Figure** **2**A–F). The apoptosis assay revealed that DLD‐1 and HCT116 cells transfected with shRNAs exhibited lower apoptotic rates than the control group (Figure 2G). Conversely, overexpression of TRIM38 in RKO and HCT116 cells resulted in a notable elevation in apoptotic rates (Figure 2H).

To identify the impact of TRIM38 on CRC cell proliferation in vivo, DLD‐1 and RKO cells were utilized to establish TRIM38 knockdown and overexpression cells via the above‐mentioned methods. Xenograft tumor models demonstrated that TRIM38 silence promoted tumor growth, as evidenced by increased tumor volume, tumor weight, and Ki‐67 level compared to the control group, while TRIM38 overexpression had the opposite effect (Figure 2I,J). Additionally, we treated C57/B6 mice with adeno‐associated virus 9 (AAV9) carrying TRIM38 (AAV9‐TRIM38) and AAV9‐vector as a negative control by means of tail vein injection. Western blot analysis of mouse colon tissues confirmed that TRIM38 expression was significantly upregulated in the AAV9‐TRIM38 group versus the AAV9‐vector group (Figure S2E, Supporting Information). Then, by establishing AOM/DSS‐induced CRC models (Figure 2K), we observed that AAV9‐TRIM38 effectively inhibited the progression of CRC, as indicated by a reduction in both tumor number and volume (Figure 2L–N). Furthermore, mice transfected with AAV9‐TRIM38 exhibited increased colorectal length, greater weight gain, and improved survival rates compared to control mice. (Figure 2O–Q). Collectively, these findings provided evidence that TRIM38 inhibits CRC cell growth and AOM/DSS‐induced tumorigenesis in mice.

### 2.4 TRIM38 Inhibits the Invasion and Metastasis of CRC Cells In Vitro and In Vivo

**Figure 3:**
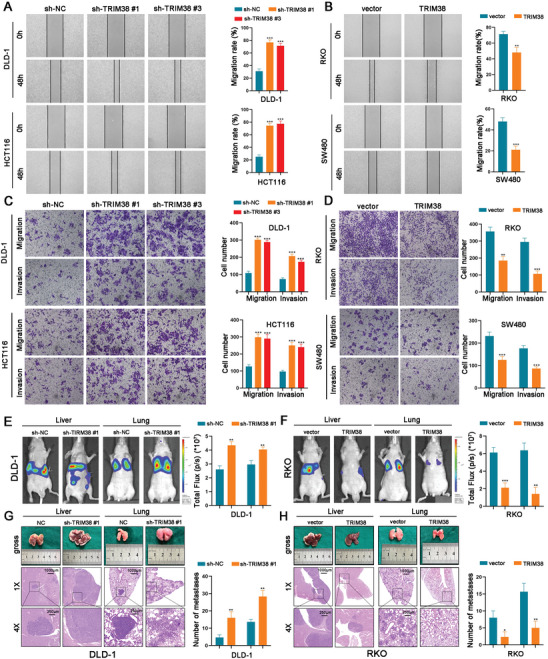
TRIM38 inhibits CRC cell invasion and metastasis in vitro and in vivo. A), B) The effect of TRIM38 on CRC cell migration ability was measured by wound‐healing assays. C,D) The effect of TRIM38 on CRC cell migration and invasion was detected by transwell assays. E,F) Representative images and analysis of fluorescence intensity for liver and lung metastasis under different TRIM38 treatments. G,H) Representative photographs and H&E staining of livers and lungs under different TRIM38 treatments. Numbers of metastasis were measured. Data are presented as mean ± SD from three independent experiments, ^*^ *P* < 0.05, ^**^ *P* < 0.01, ^***^ *P* < 0.001.

To validate whether TRIM38 affects the metastasis of CRC cells, transwell, and wound‐healing assays were performed using the above‐mentioned cells. The results demonstrated that TRIM38 silence remarkably increased the invasive and metastatic capabilities of DLD‐1 and HCT116 cells, while TRIM38 overexpression diminished the abilities of RKO and SW480 (**Figure** **3**A–D). In vivo, we established liver and lung metastasis models using the aforementioned DLD‐1 and RKO cells. TRIM38 knockdown dramatically enhanced luciferin intensity and increased the number of liver metastatic lesions as well as pulmonary (Figure 3G), while the decreased fluorescence intensity of TRIM38 and the reduction in the number of liver and lung metastatic lesions were detected after overexpression of TRIM38 (Figure 3H). Collectively, these findings suggested that TRIM38 plays a critical role in suppressing the metastasis of CRC.

### 2.5 The B30.2/SPRY Domain of TRIM38 Binds to Substrate Protein CCT6A in CRC

**Figure 4:**
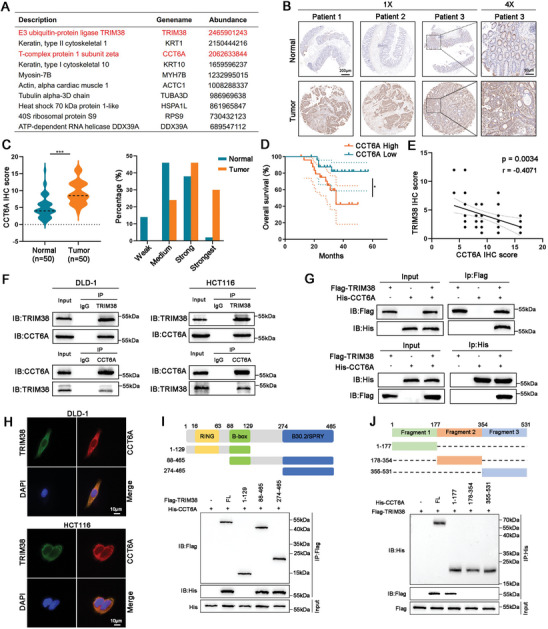
TRIM38 interacts with CCT6A in CRC cells. A) List of the top 10 differentially expressed proteins identified by mass spectrometry. B) IHC‐detected representative images of CCT6A protein level in the above‐mentioned tissue microarray. C) Analysis of H‐scores and staining degrees of CCT6A in the tissue microarray. D) Kaplan–Meier survival analysis of 50 CRC patients grouped by the CCT6A protein level in the tissue microarray. E) Correlational analysis between TRIM38 and CCT6A IHC scores in the tissue microarray. F) The endogenous interaction between TRIM38 and CCT6A in DLD‐1 and HCT116 cells was measured by Co‐IP assays. G) Co‐IP detection of the exogenous interaction between TRIM38 and CCT6A in HEK293T cells using Flag and His tag antibodies. H) Co‐localization of TRIM38 and CCT6A in DLD‐1 and HCT116 cells by immunofluorescence. I) The binding of exogenous CCT6A to various truncated TRIM38 forms in HEK293T cells measured by Co‐IP assays. J) The interaction of exogenous TRIM38 with various truncated CCT6A forms in HEK293T cells measured by Co‐IP assays. Data are presented as mean ± SD from three independent experiments, ^*^ *P* < 0.05, ^**^ *P* < 0.01, ^***^ *P* < 0.001.

To elucidate the mechanism underlying TRIM38‐mediated inhibition of CRC progression, we performed immunoprecipitation (IP) using the TRIM38 antibody and conducted mass spectrometry (MS) analysis to identify the proteins interacting with TRIM38 in CRC. The top 10 expressed proteins were listed, with CCT6A having the highest expression level among non‐cytoskeletal proteins (**Figure** **4**A; Figure S2F,G, Supporting Information). The above‐mentioned tissue microarray was analyzed using IHC, revealing the elevated expression of CCT6A protein in tumor tissues from CRC patients (Figure 4B,C). The Kaplan–Meier analysis revealed that patients with elevated CCT6A expression were correlated with a poorer prognosis (Figure 4D). More importantly, the protein level of CCT6A was inversely correlated with TRIM38 in CRC (Figure 4E). Co‐IP assays were performed to further elucidate the physical binding relationship between TRIM38 and CCT6A. In comparison to the IgG group, our results demonstrated a significant binding affinity between endogenous TRIM38 and CCT6A (Figure 4F). Additionally, transfection of plasmids encoding distinct tags for TRIM38 and CCT6A confirmed the interaction capability of exogenous TRIM38 with CCT6A (Figure 4G). Furthermore, immunofluorescence analysis revealed co‐localization of TRIM38 and CCT6A within the cytoplasm of CRC cells (Figure 4H). Then, we constructed three mutant plasmids based on the TRIM38 domain, and Co‐IP assays confirmed the interaction between CCT6A and the B30.2/SPRY domain of TRIM38 (Figure 4I). Similarly, we constructed 3 mutant plasmids based on the protein sequence of CCT6A, and the results indicated that fragment 1 of CCT6A interacted with TRIM38, which comprises the initial 177 amino acids of CCT6A protein (Figure 4J). These findings provided evidence that CCT6A is a crucial substrate for TRIM38 binding in CRC, potentially mediating the anti‐cancer activity of TRIM38.

### 2.6 TRIM38 Induces the Protein Degradation and Ubiquitination of CCT6A

**Figure 5:**
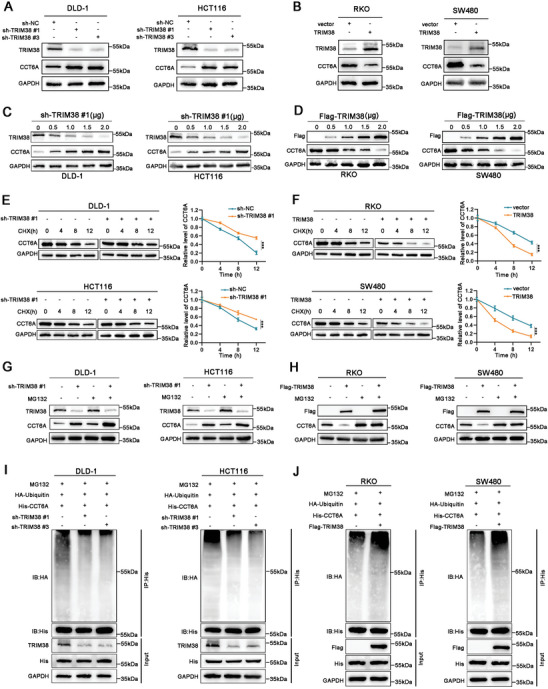
TRIM38 induces degradation and ubiquitination of CCT6A in CRC. A,B) The protein level of CCT6A in CRC cells following TRIM38 silencing or overexpression detected by western blot. C,D) Alterations in TRIM38 and CCT6A protein levels in CRC cells following treatment with varying doses of TRIM38 shRNAs and overexpression plasmids. E,F) CRC cells were treated with 100 µg mL^−1^ cycloheximide (CHX) for indicated times to assess the effect of TRIM38 on CCT6A protein stability by western blot. G,H) Indicated CRC cells were treated with proteasome inhibitor MG132 (10 µm) for 8 h, then a western blot was used to detect the reversion of MG132 on TRIM38‐mediated degradation of CCT6A. I,J) Co‐IP assays were used to measure the ubiquitination level of CCT6A influenced by TRIM38 in CRC cells. Data are presented as mean ± SD from three independent experiments, ^***^ *P* < 0.001.

We next investigated whether TRIM38 affects the expression level of CCT6A. Our results revealed that silencing TRIM38 using specific shRNAs significantly increased CCT6A protein levels in DLD‐1 and HCT116 cells (**Figure** **5**A). Conversely, overexpression of TRIM38 led to a marked reduction in CCT6A expression in RKO and SW480 cells (Figure 5B). By incrementally increasing the transfection dose of the specific shRNAs, we observed a consistent decrease in TRIM38 protein levels alongside a dose‐dependent increase in CCT6A protein levels, while overexpression of TRIM38 had the opposite result (Figure 5C,D). However, alterations in TRIM38 expression do not alter CCT6A mRNA levels, indicating that TRIM38 may regulate the stability of CCT6A protein through post‐transcriptional mechanisms (Figure S1F,G, Supporting Information). After adding Cycloheximide (CHX), a biochemical protein synthesis inhibitor, we observed that the degradation rate of CCT6A significantly decreased following TRIM38 knockdown and markedly increased upon TRIM38 overexpression in CRC cells, suggesting that TRIM38 facilitated the degradation of CCT6A (Figure 5E,F). Next, we detected the protein level of CCT6A using the proteasome inhibitor MG132. The results demonstrated that MG132 not only diminished CCT6A degradation but also impeded the impact of TRIM38 on CCT6A degradation (Figure 5G,H). Considering the E3 ligase activity of TRIM38, we observed that a reduction in TRIM38 level led to decreased ubiquitination of CCT6A, whereas an increase in TRIM38 resulted in the opposite effect (Figure 5I,J). These findings collectively indicated that TRIM38 facilitates the degradation and ubiquitination of CCT6A in CRC.

### 2.7 Poly‐Ubiquitination of CCT6A is Induced by TRIM38 via Targeting K127/K138 Residues

**Figure 6:**
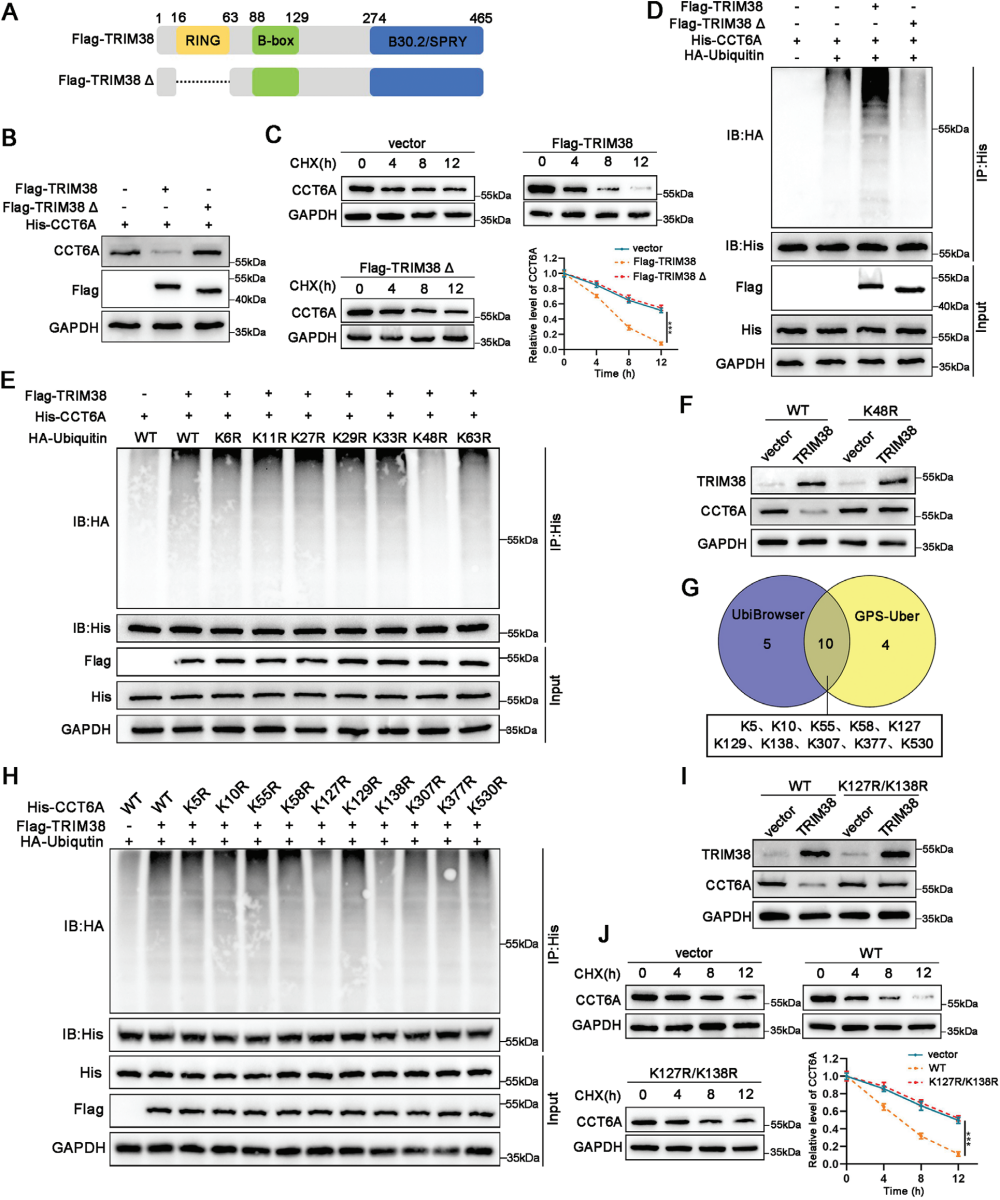
TRIM38 induces the poly‐ubiquitination and degradation of CCT6A at K127/K138 residue. A) Diagram of the TRIM38 mutant with the RING domain deleted (Flag‐TRIM38Δ). B,C) Effects of wild‐type TRIM38 and its mutant on the protein level and protein degradation of CCT6 were assessed by western blot following the indicated treatment. D) The ubiquitination level of CCT6A affected by wild‐type TRIM38 and its mutant was assessed by Co‐IP assays. E) Co‐IP assays were applied to assess the ubiquitination level of CCT6A affected by the plasmids containing seven specific ubiquitin mutants. F) The effect of the ubiquitin mutant K48R on TRIM38‐regulated CCT6A protein levels was detected by western blot. G) Potential ubiquitination sites on CCT6A predicted by UbiBrowser and GPS‐Uber database. H) The ubiquitination level of CCT6A influenced by the plasmids containing ten CCT6A mutants was measured by Co‐IP assays. I,J) Western blot was performed to assess the CCT6A mutant K127R/K138R on TRIM38‐regulated CCT6A protein level and protein degradation. All aforementioned experiments were performed in HEK293T cells. Data are presented as mean ± SD from three independent experiments, ^***^ *P* < 0.001.

In light of the enzymatic catalytic activity attributed to the RING domain, we constructed a TRIM38 mutant plasmid devoid of this domain (**Figure** **6**A). The ectopic expression of wild‐type TRIM38 significantly decreased the expression of CCT6A, whereas the mutant variant of TRIM38 was ineffective in this regard (Figure 6B). Additionally, wild‐type TRIM38 markedly increased the degradation rate of CCT6A protein, whereas the mutant variant of TRIM38 lost this capability (Figure 6C). Furthermore, wild‐type TRIM38 prominently induced polyubiquitination of CCT6A compared to its mutant counterpart (Figure 6D). These data indicated that TRIM38 induces CCT6A degradation and ubiquitination through its RING domain.

Next, we constructed seven lysine (K) to arginine (R) mutants (K6R, K11R, K27R, K29R, K33R, K48R, K63R) of ubiquitin. Our findings indicated that the K48R significantly diminished the ubiquitination of CCT6A and inhibited the reduction of CCT6A protein affected by TRIM38 (Figure 6E,F). Using the UbiBrowser and GPS‐Uber database, we identified ten potential ubiquitination sites on CCT6A by intersecting their data (Figure 6G). We then generated mutants targeting these lysine residues to validate our hypothesis. We found that only the K127R and K138R mutants blocked TRIM38‐mediated ubiquitination of CCT6A (Figure 6H). Moreover, the co‐mutations of K127 and K138 markedly abrogated the inhibitory effect of TRIM38 on CCT6A, and attenuated the degradation rate of CCT6A (Figure 6I,J). All these findings indicated that TRIM38 facilitates the K48‐linked ubiquitination of CCT6A at the K127/K138 residues.

### 2.8 TRIM38 Suppresses the Proliferation and Metastasis of CRC Mediated by CCT6A

Next, we determined whether CCT6A is a key mediator in suppressing CRC progression by TRIM38. We independently generated DLD‐1 cell lines with TRIM38 silenced, CCT6A silenced, and both TRIM38 and CCT6A co‐silenced using the specific sh‐RNAs. Similarly, we established RKO cells that overexpressed TRIM38, overexpressed CCT6A, and co‐overexpressed both CCT6A and TRIM38 via the plasmids. The CCK‐8 and plate colony formation assays revealed that silencing CCT6A not only inhibited the proliferation of DLD‐1 cells but also diminished the enhancement of cell proliferation induced by TRIM38 knockdown (Figure S3A–C, Supporting Information). Conversely, overexpression of CCT6A exerted an opposing effect (Figure S3B–D, Supporting Information). Next, transwell and wound‐healing assays revealed that silencing CCT6A markedly diminished the invasive and metastatic capabilities of DLD‐1 cells, while also attenuating the effect of TRIM38 silencing in enhancing CRC cell invasion and metastasis, and vice–versa (Figure S3E–H, Supporting Information). In vivo, the xenograft model demonstrated that downregulation of CCT6A significantly inhibited tumor growth and negated the influence of TRIM38 knockdown on tumor progression (Figure S4A–C, Supporting Information). The liver metastasis model further confirmed that CCT6A knockdown disrupted the promotion of liver metastasis induced by TRIM38 silencing (Figure S4D,E, Supporting Information). These findings underscored the critical role of TRIM38 in facilitating CRC progression through its mediation of CCT6A.

### 2.9 TRIM38/CCT6A Regulates the MYC Pathway to Affect CRC Progression

**Figure 7:**
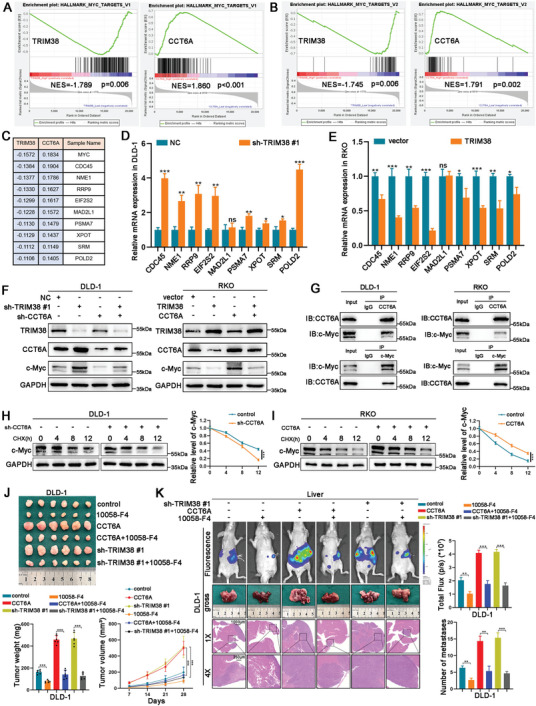
TRIM38/CCT6A regulates CRC progression by the MYC pathway. A), B) The relationship between the MYC signaling pathway and TRIM38 as well as CCT6A in GSEA analysis based on TCGA database. C) For the GSEA analysis of TRIM38, the top 10 genes with the highest rank metric scores in the MYC pathway related to downregulated TRIM38 were listed, along with their scores from the GSEA analysis of CCT6A upregulation. D,E) The mRNA expression of MYC pathway‐related genes in DLD‐1 and RKO cells were assessed by qRT‐PCR after TRIM38 knockdown or overexpression. F) The effects of alterations in the expression of TRIM38 and CCT6A on the c‐Myc protein level in DLD‐1 and RKO cells were assessed by western blot. G) The endogenous interaction between CCT6A and c‐Myc in DLD‐1 and RKO cells was detected by Co‐IP assays. H, I) Following CHX treatment, the effect of CCT6A on c‐Myc protein stability was measured by western blot. J,K) Following 10058‐F4 treatment, the effects on tumor growth and metastasis mediated by TRIM38 and CCT6A were evaluated by both the xenograft and liver metastasis models. Data are presented as mean ± SD from three independent experiments, ^*^ *P* < 0.05, ^**^ *P* < 0.01, ^***^ *P* < 0.001.

**Figure 8:**
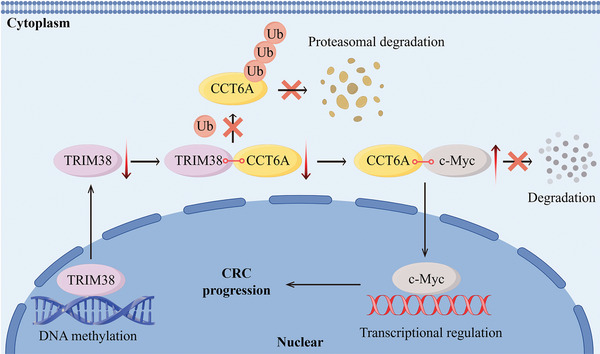
Schematic for the mechanism of TRIM38/CCT6A/c‐Myc axis in facilitating tumor progression in CRC.

To elucidate the impact of TRIM38‐mediated CCT6A ubiquitination on CRC progression, we conducted a bioinformatics analysis utilizing the TCGA database. The results from Gene Set Enrichment Analysis (GSEA) indicated a significant correlation between MYC pathway activation and reduced TRIM38 expression alongside elevated CCT6A levels, corroborated by two independent gene sets associated with the MYC pathway (**Figure** **7**A,B). For the V1 gene sets associated with the MYC pathway, we identified the top 10 genes linked to downregulated TRIM38 based on their rank metric scores. Our analysis revealed a strong correlation between these genes and CCT6A upregulation in GSEA assessments (Figure 7C). Subsequently, we quantified the mRNA levels of these genes through qRT‐PCR following either TRIM38 knockdown or overexpression, and our findings were highly consistent with those obtained from GSEA analysis (Figure 7D,E). Next, we evaluated the protein level of c‐Myc by western blot, demonstrating that TRIM38 exerted a negative regulatory influence on c‐Myc protein expression, while CCT6A displayed a positive regulatory effect. Importantly, the modulation of c‐Myc by TRIM38 was dependent on CCT6A (Figure 7F). Co‐IP assays further revealed a binding relationship between CCT6A and c‐Myc (Figure 7G). Following CHX treatment, we observed that CCT6A reduced the degradation rate of the c‐Myc protein and enhanced its protein stability (Figure 7H,I). To explore the role of c‐Myc in CRC progression, we utilized a specific c‐Myc inhibitor, 10058‐F4. Both colony formation and transwell assays revealed that 10058‐F4 effectively restrained the proliferation, invasion, and metastasis of CRC cells (Figure S5A,B, Supporting Information). Furthermore, 10058‐F4 exhibited an inhibitory effect on the oncogenic functions associated with TRIM38 silencing and CCT6A overexpression in vitro. Similar results were further confirmed in vivo through both the subcutaneous xenograft and liver metastasis models (Figure 7J,K). Taken together, our findings confirmed that the TRIM38/CCT6A/c‐MYC axis is a key driver of CRC progression (**Figure** **8**).

## 3 Discussion

TRIM38 is a typical TRIM protein with a C‐terminal PRY‐SPRY (B30.2) domain, widely expressed in various cell types of humans and mice, and plays a crucial role in numerous biological processes, including cardiac fibrosis, innate immunity, inflammatory response, and bone remodeling.^[^ ^29^, ^30^, ^31^, ^32^ ^]^ Nevertheless, limited research has uncovered the pivotal function of TRIM38 in tumorigenesis. Recently, research demonstrated that TRIM38 interacts with GLUT1 to suppress the progression of bladder cancer,^[^ ^33^ ^]^ and another study revealed that TRIM38 regulates the AMPK/NF‐κB/NLRP3 pathway to inhibit the progression of lung cancer,^[^ ^34^ ^]^ gradually revealing the importance of TRIM38 in malignancy. However, the role of TRIM38 in CRC remains completely unknown. In this study, we first reported that TRIM38 expression was remarkably downregulated in CRC, which was closely associated with unfavorable clinical features and prognosis. Functionally, our findings indicated that the TRIM38 downregulation promotes the tumorigenesis, proliferation, invasion, and metastasis of CRC. DNA methylation is a prevalent chemical modification wherein specific bases within a DNA sequence undergo methylation catalyzed by DNA methyltransferase (DNMT), mainly targeting CpG islands in gene expression regulatory elements.^[^ ^35^ ^]^ Abnormal CpG methylation reportedly results in the silencing of tumor‐suppressor genes and the activation of oncogenes, which are intricately linked to tumorigenesis and cancer progression.^[^ ^36^, ^37^ ^]^ Based on website and bioinformatics data analysis, we identified the potential methylation of CpG islands in the promoter region of TRIM38. To validate this hypothesis, we performed BSP, MSP, and 5‐Aza treatment, which verified that the silencing of TRIM38 was associated with promoter CpG hypermethylation in CRC.

By applying MS analysis and Co‐IP assays, we identified CCT6A as a downstream binding protein of TRIM38 in CRC. As a critical submit of the CCT complex, CCT6A plays a carcinogenic role in various malignancies.^[^ ^38^, ^39^, ^40^, ^41^ ^]^ For example, CCT6A promotes tumor progression in lung adenocarcinoma through the STAT1/HK2 axis,^[^ ^41^ ^]^ and facilitates metastasis in non‐small‐cell lung carcinoma by competitively binding to SMAD2.^[^ ^39^ ^]^ In CRC, it has been reported that CCT6A is highly expressed in tumor tissues and serves as a predictor of poor prognosis.^[^ ^42^ ^]^ Silencing CCT6A can effectively inhibit the proliferation, invasion, and metastasis of CRC cells.^[^ ^43^ ^]^ These results are coincident with our study. Moreover, we found that the protein level of CCT6A exhibited a negative correlation with TRIM38 in CRC tissues, suggesting an inverse regulation of CCT6A by TRIM38. Mechanistically, we further elucidated that the SPRY domain of TRIM38 interacts with CCT6A, facilitating the K48‐linked polyubiquitination of CCT6A via targeting K127 and K138 residues, which is catalyzed by the RING domain of TRIM38.

The MYC gene family is a potent driver of various human cancers and can regulate numerous biological activities that contribute to tumor progression.^[^ ^44^, ^45^ ^]^ As the most critical oncogene in the MYC protein, c‐Myc is crucial in modulating proliferation, invasion, cell survival, genomic instability, angiogenesis, metabolism, and immune evasion via both transcriptional activation and repression.^[^ ^46^ ^]^ Amplification of c‐Myc has been observed in CRC, and its role in promoting CRC growth and metastasis has been extensively demonstrated.^[^ ^47^, ^48^ ^]^ Despite its importance in carcinogenesis, c‐Myc is highly unstable and easily degraded via the ubiquitin‒proteasome pathway.^[^ ^49^ ^]^ Notably, previous studies have indicated that CCT6A primarily works in tumors through the following mechanisms: protein folding regulation, competitively binding to client proteins, and enhancing the stability of client proteins.^[^ ^39^, ^41^ ^]^ GSEA analysis in the present study demonstrated that silencing TRIM38 and upregulating CCT6A are closely associated with MYC pathway activation. At the protein level, our findings indicated that TRIM38 could suppress c‐Myc expression by degrading CCT6A. Additionally, CCT6A was observed to interact with c‐Myc and reduce its degradation. Finally, evidence using 10058‐F4 demonstrated that the TRIM38/CCT6A/c‐myc axis could inhibit CRC proliferation and metastasis.

We recognize that our study has several limitations that warrant further consideration. First, although we have established that the downregulation of TRIM38 in CRC is attributable to DNA methylation, the precise underlying mechanisms remain unclear. Second, the mechanism by which CCT6A inhibits c‐Myc degradation merits additional exploration. Third, we did not investigate the downstream signaling pathways and molecules regulated by c‐Myc in detail. We will further explore these aforementioned questions in subsequent experiments.

In conclusion, our study demonstrates that TRIM38 suppressed the progression of CRC in both clinical features and cell malignant phenotype. Mechanistically, DNA hypermethylation was responsible for TRIM38 downregulation. Silencing TRIM38 reduces CCT6A degradation via the ubiquitination pathway, leading to increased stability of c‐Myc mediated by CCT6A. These findings unveil a novel mechanism for protein regulation in CRC, offering a potential therapeutic target for the disease.

## 4 Experimental Section

### Human Specimens

A total of 200 tumor samples and paired adjacent normal tissues were collected from patients diagnosed with CRC between 2017 and 2018 at the First Affiliated Hospital of Nanjing Medical University. Fresh tissues were immediately frozen at −80 °C after surgery for long‐term preservation. This study was ratified by the Human Ethics Committee of the First Affiliated Hospital of Nanjing Medical University (2019‐SRFA‐ 018). Each patient was informed about the study and signed a consent form. Patients who have received neoadjuvant chemotherapy, radiotherapy, or immunotherapy were excluded.

### Cell Lines and Culture

Human normal colonic epithelial cell line NCM460, human CRC cell lines (DLD‐1, HCT116, RKO, SW480, SW620, HT29, CACO2), and HEK293T were obtained from the Cell Bank of Type Culture Collection of the Chinese Academy of Sciences (Shanghai, China). The cells were cultured in the recommended medium with 10% fetal bovine serum at a 37 °C in a 5% CO2 humidified incubator.

### Quantitative Real‐Time PCR (qRT‐PCR)

Total RNA was extracted from tissues and cells by TRIzol reagent (Invitrogen, USA). RNA was reverse transcribed into cDNA by the PrimeScript RT reagent kit (Takara, Dalian, China). The specific primers for qRT‐PCR in the study were shown in Table S1 (Supporting Information). The detailed procedure was referred to the publication.^[^ ^50^ ^]^

### Immunohistochemistry (IHC)

IHC was performed as previously described.^[^ ^50^ ^]^ The staining intensity and proportion were used to evaluate the protein levels of TRIM38 and CCT6A. The staining intensity was graded as 1 (weak), 2 (medium), 3 (strong), and 4 (strongest). The proportion was categorized as 1 (1–25%), 2 (26–50%), 3 (51–75%), and 4 (76–100%). The IHC score was multiplied by staining intensity and proportion. All the primary antibodies were listed in Table S2 (Supporting Information).

### Cell Transfection

The short hair RNAs (shRNAs) of TRIM38 and CCT6A were synthesized by Genomeditech (Shanghai, China). The overexpression plasmids, including Flag‐TRIM38, His‐CCT6A, and HA‐ubiquitin, were obtained from Genomeditech. A series of truncated or mutated plasmids were obtained from Obio (Shanghai, China). The above shRNAs and plasmids were transfected by Lipofectamine 3000 (Invitrogen). The sequences of shRNAs were listed in Table S1 (Supporting Information).

### Cell Proliferation Assays

The ability of cell colony formation was measured by plate colony formation assay. Cell viability was detected by Cell Counting Kit‐8 assay (Dojindo, Japan). DNA replication activity was observed by 5‐ethynyl‐2‐deoxyuridine (EdU, Beyotime, Shanghai, China) assay. Detailed methods of the above assays were performed as described previously.^[^ ^51^ ^]^

### Transwell and Wound‐Healing Assays

The invasion and migration ability of CRC cells in vitro were detected by transwell assay and wound‐healing assays as described previously.^[^ ^52^ ^]^

### Bioinformatics Analysis

CpG islands in the promoter region of TRIM38 were analyzed using the MethPrimer tool (https://www.urogene.org/methprimer/), which is designed to identify CpG islands within a given DNA sequence. Methylation and mRNA expression data for TRIM38 in CRC were retrieved from cBioPortal, a comprehensive web‐based platform providing access to various cancer genomics datasets.

### Methylation‐Specific PCR (MSP)

TIANamp Genomic DNA Kit (TIANGEN, Beijing, China) was used to extract genomic DNA from clinical tissues following the manufacturer's protocol. DNA incubated with sodium bisulfite was treated with a Methylation‐Gold Kit (Zymo, USA). Primers for methylated (M) and unmethylated (U) sequences are listed in Table S1 (Supporting Information).

### Bisulfite Sequencing PCR (BSP)

The genomic DNA was bisulfite treated using EpiTect Fast DNA Bisulfite Kit (QIAGEN, Germany). The modified DNA was PCR‐amplified by primers of BSP, and the products were cloned into pMD19‐T (TaKaRa). After amplification of the cloned PCR fragments, the cloned products were sent for DNA sequencing. The forward and reverse primers for BSP are listed in Table S1 (Supporting Information).

### Co‐Immunoprecipitation (Co‐IP) Assay

Cell lysates were obtained after centrifugation by using IP lysis buffer (Beyotime, Shanghai, China). Then cell lysates were incubated with the specific primary antibody at 4 °C overnight to form immune complexes. After activating A/G magnetic beads by IP lysis buffer, immune complexes were incubated with A/G magnetic beads for 2 h. Then A/G magnetic beads were washed twice with IP lysis buffer, followed by cleaning once with pure water. The collected A/G magnetic beads supplemented with 1 × SDS loading buffer were boiled for 10 min. The immunoprecipitated proteins were used for mass spectrometry analysis and western blot. The primary antibodies were listed in Table S2 (Supporting Information).

### Ubiquitination Assay

Cells transfected with specific shRNAs or plasmids were treated with MG132 for 8 h and collected. The level of CCT6A ubiquitination was analyzed by immunoblotting with an antibody against HA.

### 5‐Aza‐2′‐Deoxycytidine (5‐Aza) Treatment

Cells were cultured to a density of 30%, and varying doses of 5‐Aza (Sigma, MO, USA) were administered for treatment. The 5‐Aza was replenished daily over a period of four consecutive days. Subsequently, proteins were extracted from the treated cells for immunoblotting analysis.

### Western Blot

Proteins were separated by 10% or 12.5% SDS‐PAGE (Beyotime, Shanghai, China) and transferred to PVDF membranes (Roche, Shanghai, China). Then the protein bands were blocked in protein free rapid blocking buffer (Epizyme, Shanghai, China) for 20 min, and incubated with the specific primary antibody overnight at 4 °C. Next, the bands were incubated with the secondary antibody for 2 h, and imaged using a chemiluminescence detection reagent (Epizyme) by a bio‐imaging system. The primary antibodies for western blot are listed in Table S2 (Supporting Information).

### Cell Apoptosis Analysis

Cells treated with shRNAs or plasmids were collected by binding buffer and mixed with an Annexin V‐FITC Apoptosis Detection Kit I (MUTI SCIENCES, Hangzhou, China) in flow cytometry tubes. The rate of apoptotic cells was analyzed using BD FACSCanto II.

### Immunofluorescence (IF)

Cells were fixed with an immunostaining fixative (P0098, Beyotime) and washed with an immunostaining detergent (P0106, Beyotime). Then the cells were blocked with a blocking buffer (P0102, Beyotime) and incubated overnight at 4 °C with the primary antibody. Next, the cells were treated with the Alexa Fluo labeled secondary antibodies in the dark. DAPI was used to visualize the nuclei.

### Animal Models

All the animal experiments were approved by the Institutional Animal Care and Use Committee of Nanjing Medical University (IACUC‐2407096). Five‐week‐old male BALB/c nude mice were obtained from the Animal Center of Nanjing Medical University for the establishment of subcutaneous tumor xenograft, liver metastasis, and lung metastasis models. For the xenograft model, 1 × 10^6^ transfected DLD‐1 and RKO cells were subcutaneously injected into the right armpits of the mice. Tumor volume was measured weekly. All the mice were sacrificed four weeks post‐injection, after which tumors were excised and subjected to H&E and IHC staining. For the liver and lung metastasis models, 5 × 10^5^ luciferase‐labeled transfected cells were injected into the splenic tip and tail vein, respectively. After four weeks, all the mice received an intraperitoneal injection of D‐luciferin (Goldbio, USA), and were imaged 10 min later with an IVIS 100 Imaging System (Xenogen, Hopkinton, MA, USA) for biological imaging. Then liver and lung tissues were dissected and H&E staining was performed. For c‐Myc inhibitor treatment in the both xenograft and liver metastasis model, mice were intravenously injected with 10058‐F4 (15 mg kg^−1^) three times per week during the first two weeks, followed by two injections per week during the last two weeks, for a total of 10 injections. For AOM/DSS colorectal tumorigenesis models, the adeno‐associated viral vector carrying full‐length cDNA of TRIM38 (AAV9‐TRIM38, Obio, Shanghai, China), and its control (AAV9‐vector) were injected into C57/B6 mice via tail vein. One week post‐injection, all mice received an intraperitoneal dose of azoxymethane (AOM; Sigma) at 10 mg kg^−1^ body weight. After one week, 2.5% dextran sulfate sodium (DSS, MP Biologicals) was given in the drinking water for 1 week followed by normal water for 2 weeks. This cycle was repeated three times. Then all mice were sacrificed to resect their colons for subsequent H&E staining.

### Statistical Analysis

All statistical analyses were performed using SPSS 22.0 (Chicago, IL, USA) and GraphPad Prism 9.0 (La Jolla, CA, USA). Each experiment was repeated at least three times. Data are shown as the mean ± standard deviation. *P* < 0.05 is considered statistically significant.

## Conflict of Interest

The authors declare no conflict of interest.

## Supporting information



Supporting Information

## Data Availability

The data that support the findings of this study are available from the corresponding author upon reasonable request.
