# Supplementary material for: TRIM38 Suppresses the Progression of Colorectal Cancer via Enhancing CCT6A Ubiquitination to Inhibit the MYC Pathway
Source: Adv Sci (Weinh). 2025 Mar 6;12(16):2411285. doi: 10.1002/advs.202411285 (PMC12021106; doi:10.1002/advs.202411285)

**Table S1.** Sequences of qRT-PCR primers, shRNAs, MSP and BSP primers in this study.

**Table S2.** The information of antibodies used in this study.

**Fig. S1. Related detection of TRIM38 mRNA expression. A, B** The mRNA expression of TRIM38 in the GEO datasets and TCGA database. **C** The correlation analysis of TRIM38 mRNA expression and methylation level in cBioPortal database. **D, E** The efficiency of TRIM38 knockdown and overexpression in selected CRC cells was detected by qRT-PCR. **F, G** The mRNA expression of CCT6A after TRIM38 knockdown or overexpression in selected cells. Data are presented as mean ± SD from three independent experiments, ^**^P < 0.01, ^***^P < 0.001.

**Fig. S2. Related detection of TRIM38 protein level and protein identification. A, B** The complete staining results of TRIM38 and CCT6A by IHC staining in the tissue microarray. **C, D** The efficiency of TRIM38 knockdown and overexpression in selected CRC cells was assessed by western blot at the protein level. **E** Western blot of TRIM38 expression in AAV9-vector and AAV9-TRIM38 colon tissues. **F, G** The identified TRIM38 and CCT6A were shown by mass spectrometry analysis.

**Fig. S3. TRIM38 suppresses CRC cell proliferation and metastasis in vitro via CCT6A. A-D** Following the indicated treatment, the impact of CCT6A on TRIM38-mediated inhibition of CRC cell proliferation ability in CRC cells was measured by CCK-8 assays and colony formation assay. **E-H** The effect of CCT6A on TRIM38-mediated suppression of CRC cell invasion and metastasis in CRC cells was evaluated by transwell assays and wound-healing assays. Data are presented as mean ± SD from three independent experiments, ^**^P < 0.01, ^***^P < 0.001.

**Fig. S4. TRIM38 inhibits tumor growth and metastasis of CRC *in vivo* reliant on CCT6A. A-C** The xenograft model was applied to evaluate the effect of CCT6A on TRIM38-mediated inhibition of tumor growth *in vivo* following the indicated treatment, shown by tumor volume and tumor weight. **D, E** The liver metastasis model was used to assess the impact of CCT6A on TRIM38-mediated suppression of tumor metastasis, exhibited by fluorescence intensity and number of metastatic lesions. Data are presented as mean ± SD from three independent experiments, ^**^P < 0.01, ^***^P < 0.001.

**Fig. S5. 10058-F4 inhibits CRC cell proliferation and metastasis mediated by TRIM38/CCT6A *in vitro*.** DLD-1 and RKO cells were pretreated with c-Myc specific inhibitor 10058-F4 for 48 hours. **A** The effect of 10058-F4 on cell proliferation mediated by TRIM38/CCT6A was assessed using plate colony formation assays. **B** The effect of 10058-F4 on cell invasion and metastasis mediated by TRIM38/CCT6A was evaluated through transwell assays. Data are presented as mean ± SD from three independent experiments, ^***^P < 0.001.

**Table S1.** Sequences of qRT-PCR primers, shRNAs, MSP and BSP primers in this study.

| **Gene name** | **Forward primer (5'->3')** | **Reverse primer (5'->3')** |
| --- | --- | --- |
| TRIM38 | TGAGGATCGGAGACAAGTG | AGCCTTCACAACCCAAGA |
| CCT6A | ATTGAAAGCATCCCTGTTGGTA | CTTTGTGGCGGGAGCATTTT |
| CDC45 | GAACACACTCTCCGTGGACT | TGAACCTGGCTGCGGTATAG |
| NME1 | GGGCAGACCACATTGCTTTT | ATGGTCGGGGATGGTAACAC |
| RRP9 | GGGATGGGACTGTACGTGTG | GCTTCTTGGAGAGACCCCAC |
| EIF2S2 | AAACTGAACAATGGCAGGCG | CTCAAGGTCCCACCACACTC |
| MAD2L1 | ACGGTGACATTTCTGCCACT | TGGTCCCGACTCTTCCCATT |
| PSMA7 | GTGTGCGCTTTTGAGAGTCG | TCTTCCTCGAACACCAACCG |
| XPOT | TTCACAGTTGAACCTCAGCAC | CTGCTCCGAACTTTTGCACT |
| SRM | AAGATGGTGTCCTCTGCTGC | AAGTTCGTGCTCGGGTTCTT |
| PLOD2 | CCAACCCCTTTTCTACCTCGG | TTTCTGGCTTCCGCTTGACT |
| GAPDH | GGACCTGACCTGCCGTCTAG | GTAGCCCAGGATGCCCTTGA |

1. **List of primers in the study**
2. **List of short hairpin RNAs (shRNAs) sequence**

| sh-TRIM38 #1 | Sense | 5’-GCACCAAGAAGATGATGGAGG-3’ |
| --- | --- | --- |
|  | Antisense | 5’-CCTCCATCATCTTCTTGGTGC-3’ |
| sh-TRIM38 #2 | Sense | 5’-AGCTCTTGTTGAAGACGTATG-3’ |
|  | Antisense | 5’-CATACGTCTTCAACAAGAGCT-3’ |
| sh-TRIM38 #3 | Sense | 5’-AGATACAGCTCATCACGAACT-3’ |
|  | Antisense | 5’-AGTTCGTGATGAGCTGTATCT-3’ |
| sh-CCT6A | Sense | 5’- AAGGATTTGTTGTTATTAATC-3’ |
|  | Antisense | 5’- GATTAATAACAACAAATCCTT-3’ |

1. **MSP primers**

| TRIM38 | Methylated Forwad Primer | 5’-TAGTAATAGAGGGGATTAGTAGCGA-3’ |
| --- | --- | --- |
|  | Methylated Reverse Primer | 5’-ATTAAAAAAACCCTACTATCACGAA-3’ |
|  | Unmethylated Reverse Primer | 5’-AGTAATAGAGGGGATTAGTAGTGA-3’ |
|  | Unmethylated Reverse Primer | 5’-ATTAAAAAAACCCTACTATCACAAA-3’ |

1. **BSP primers**

| TRIM38 | Forward primer | 5’- ATATTTTGTGTTGGGGTTATATGATAG-3’ |
| --- | --- | --- |
|  | Reverse primer | 5’-TCAAAATTAAAAAAACCCTACTATCAC-3’ |

| **Antibody** | **Manufacturer** | **Application** |
| --- | --- | --- |
| TRIM38 | proteintech: 13405-1-AP | 1:1000 for WB, 1:100 for IHC,1:100 for IP |
| TRIM38 | ThermoFisher: MA5-26235 | 1:100 for IF |
| CCT6A | proteintech: 19793-1-AP | 1:1000 for WB, 1:100 for IF,1:100 for IP |
| CCT6A | Abcam: ab110905 | 1:1000 for IHC |
| c-Myc | proteintech:67447-1-Ig | 1:10000 for WB, 1:100 for IP |
| GAPDH | proteintech:60004-1-Ig | 1:50000 for WB |
| Ub | proteintech:10201-2-AP | 1:5000 for WB |
| Ki-67 | Abcam: ab15580 | 1:500 for IHC |
| Flag | CST: #14793 | 1:1000 for WB; 1:50 for IP |
| His | CST: #12698 | 1:1000 for WB, 1:50 for IP |
| HA | CST: #3724 | 1:1000 for WB |

**Table S2:** List of antibodies in the study.


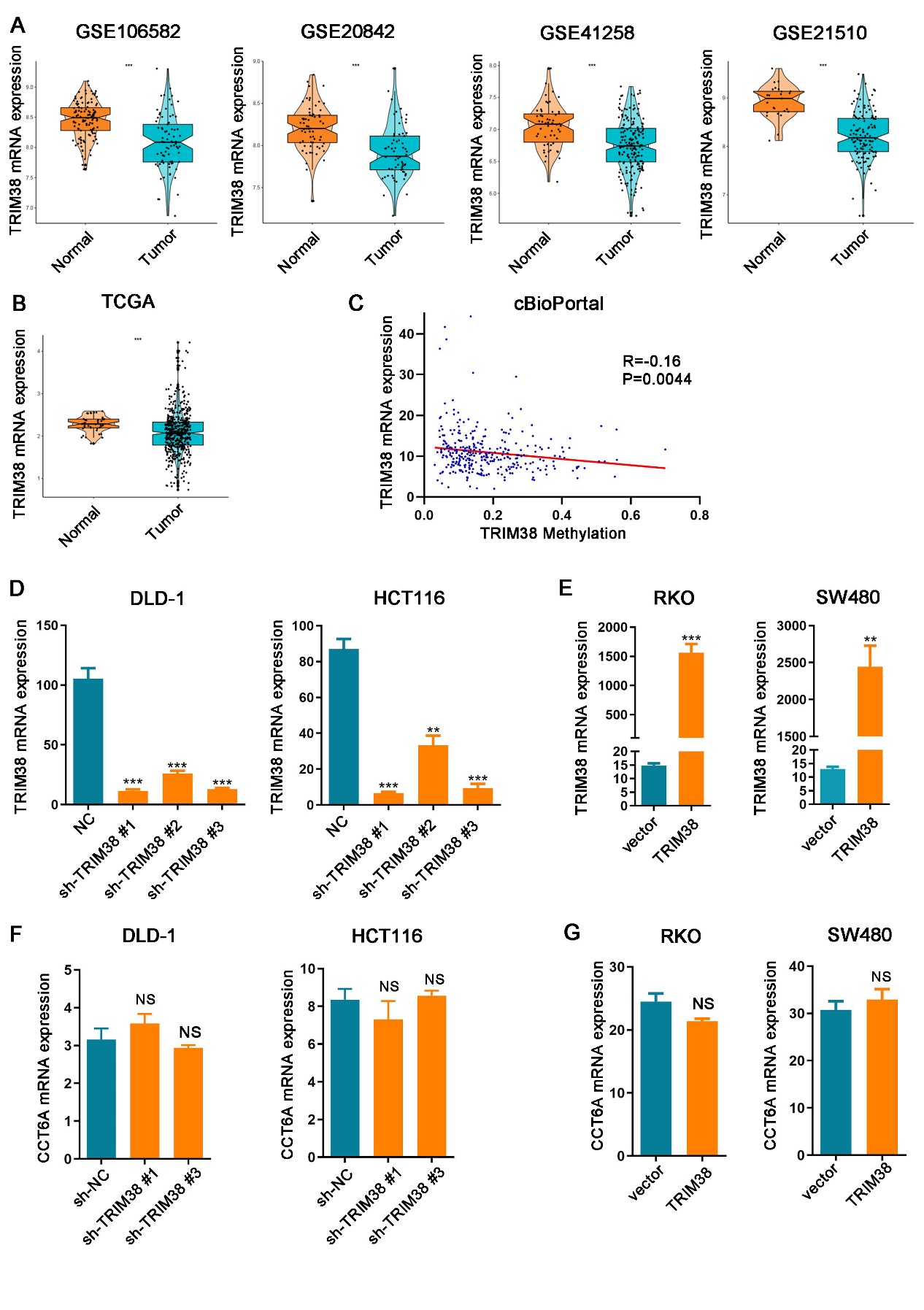
**Fig. S1**


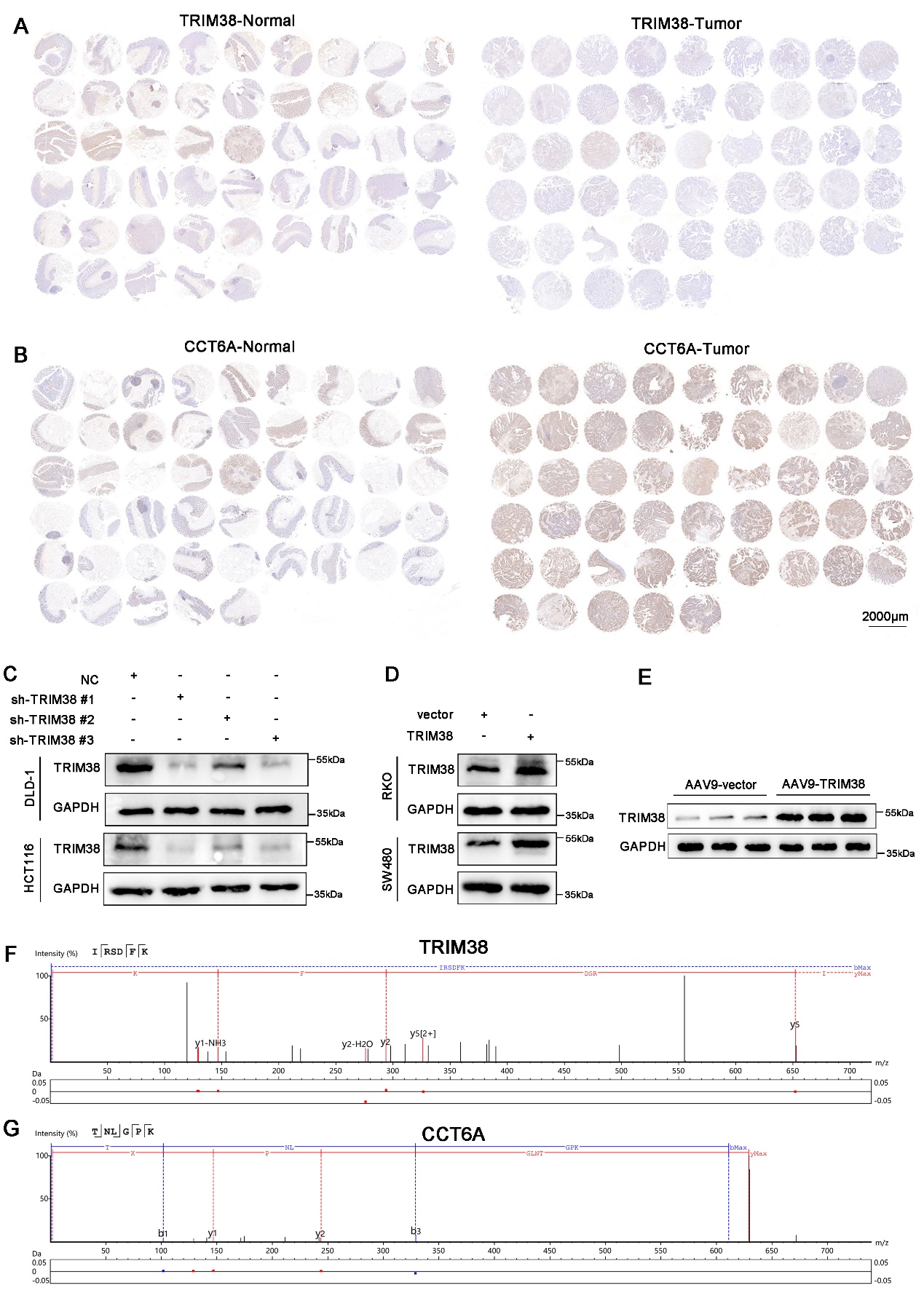
**Fig. S2**


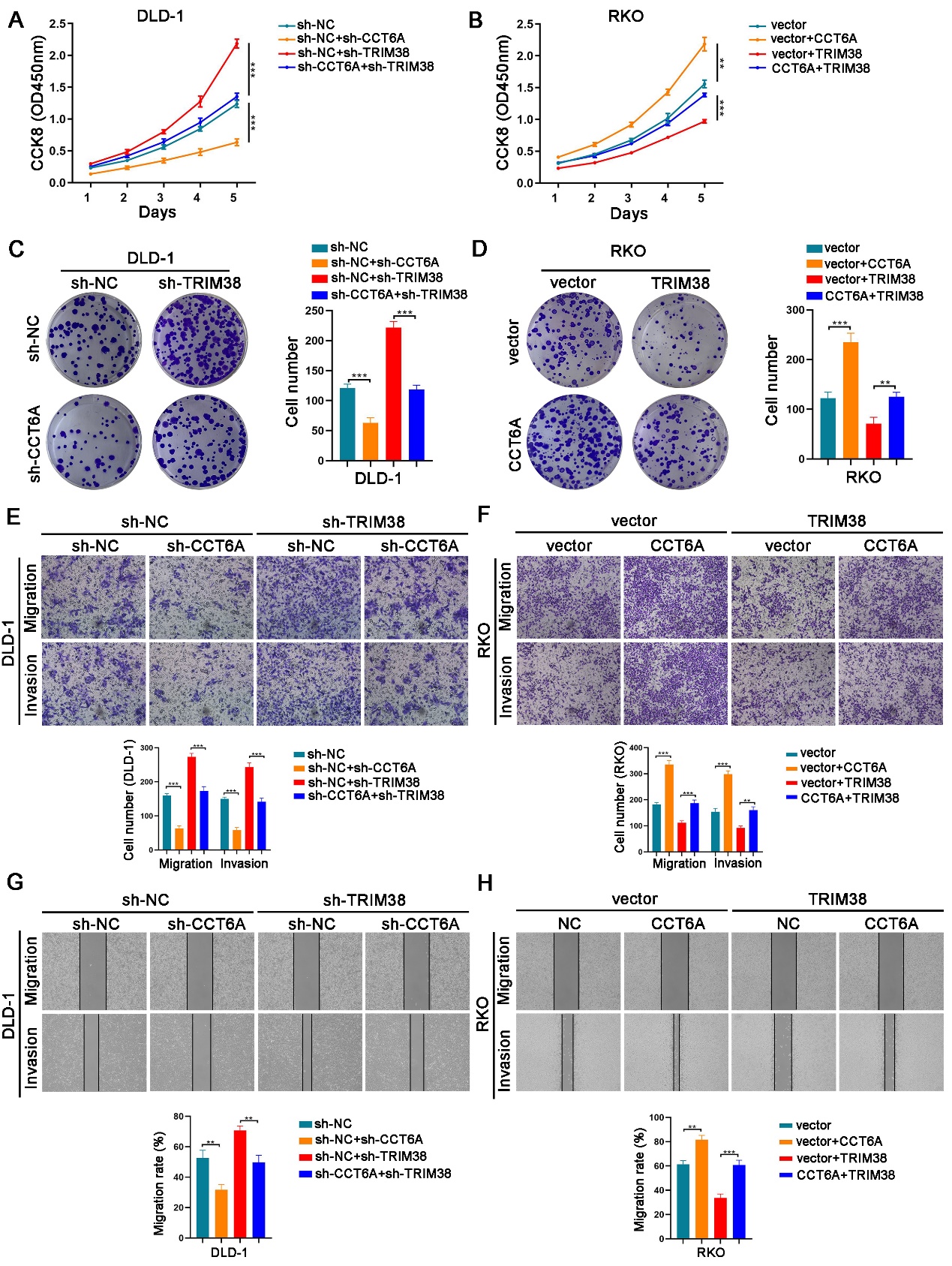
**Fig. S3**


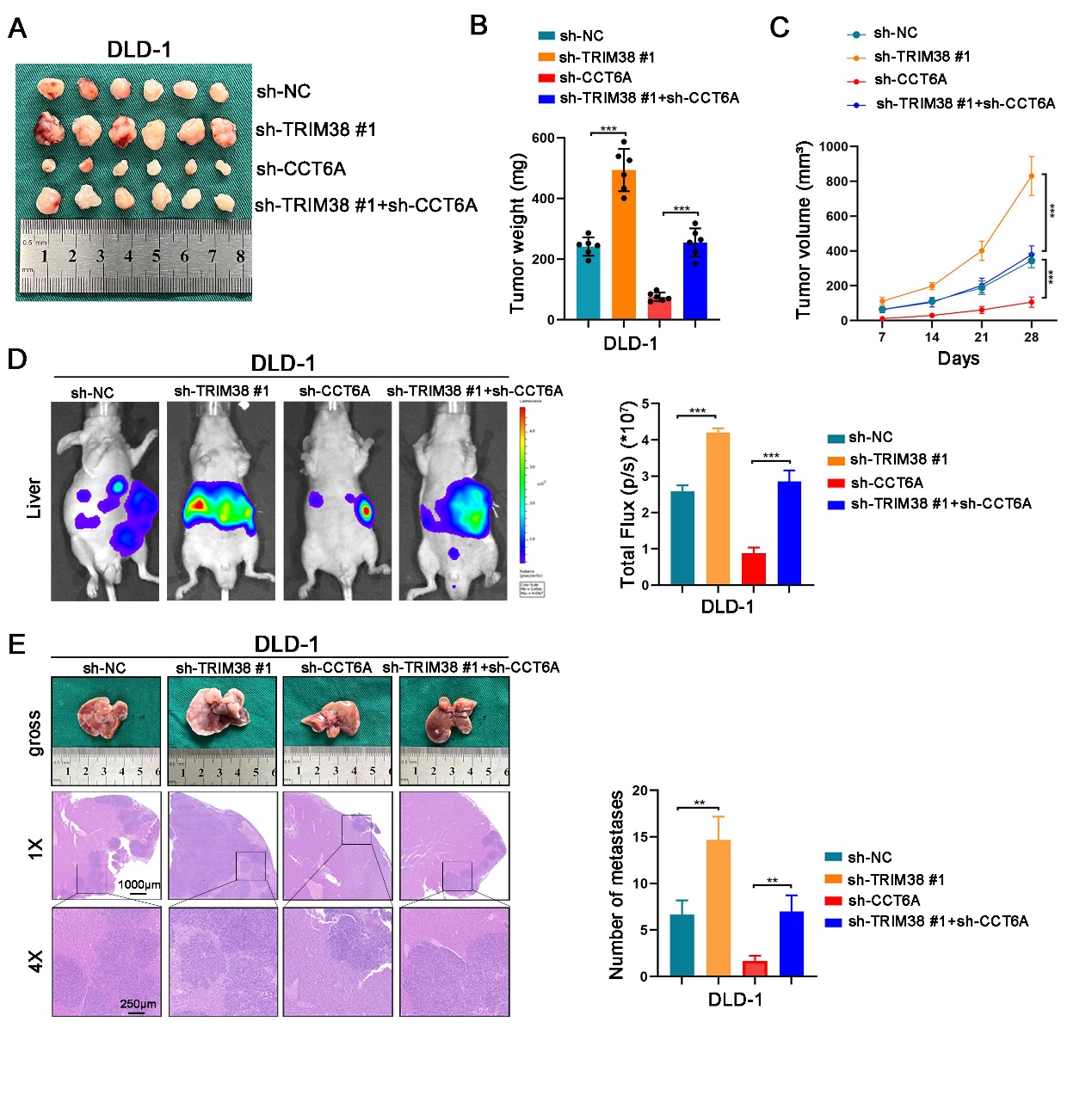
**Fig. S4**

**Fig. S5**


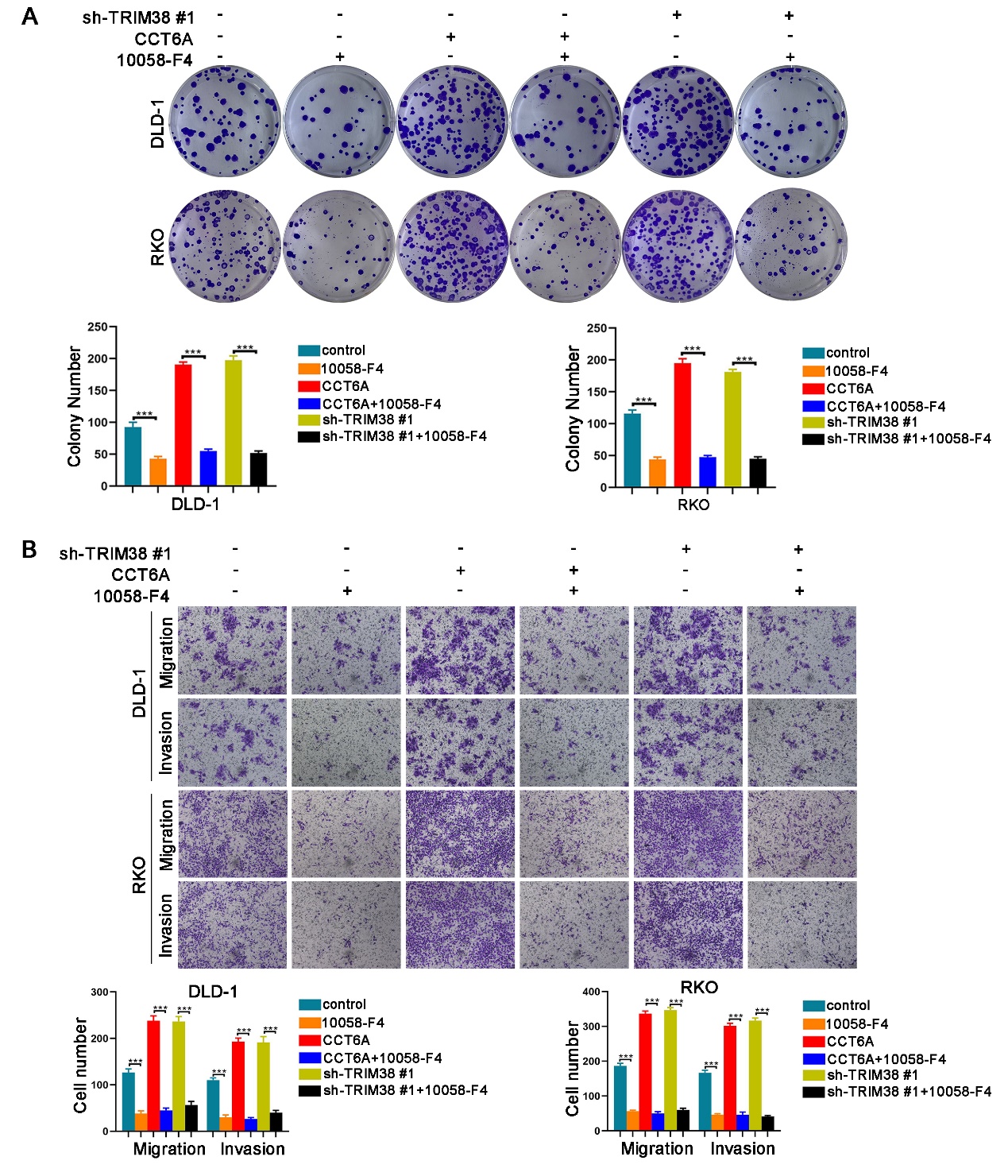

Supplement: Supplementary file 1 — Supporting Information [file ADVS-12-2411285-s001.docx]
